# A Disclaimer

**Published:** 1912-03-09

**Authors:** H. Percy Adams, Charles Holden

**Affiliations:** 28 Woburn Place. Russell Square, W.C.; 28 Woburn Place. Russell Square, W.C.


*To the Editor of* The Hospital.

### COMPETITIONS FOR HOSPITAL BUILDINGS: A Disclaimer.

*To the Editor of* The Hospital.

Sir,—With reference to the articles on the above which appeared in your issue of March 2, 1912, in which your correspondent severely criticised the first and second premiated designs, and stated that our design, placed third, is distinctly superior to the other two, we should like to state that we have no idea who was the author of these articles, and to dissociate ourselves from them. Whether one agrees or not with an assessor's decision, we have always accepted it without complaint.—Faithfully yours,

H. Percy Adams and Charles Holden.

28 Woburn Place.

Russell Square, W.C., March 5.

